# Effect of N-acetylcysteine and allopurinol combination to protect spinal cord ischemia/reperfusion injury induced by aortic cross-clamping in rat model

**DOI:** 10.1186/s13019-015-0284-z

**Published:** 2015-07-08

**Authors:** Bilgehan Erkut, Oruc Alper Onk

**Affiliations:** Department of Cardiovascular Surgery, Erzincan University Medical Faculty, Training and Research Hospital, Erzincan, Turkey

**Keywords:** Spinal cord ischemia, N-acetylcysteine, Allopurinol, Aortic cross-clamp, Paraplegia, Ischemia/reperfusion injury

## Abstract

**Purpose:**

The aim of this experimental study was to determine whether combination of N-acetylcysteine and allopurinol can reduce the ischemia/reperfusion injury of spinal cord in a rat model.

**Methods:**

Twenty-seven Spraque Dawley rats, all male, weighing between 220 to 370 (mean 325) gr were used in the study. 27 rats were divided into three groups: sham group, control group and experimental group. Abdominal aortic occlusion between the renal arteries and iliac bifurcations was carried out for 60 min with proximal and distal clip in control and experimental groups. Hindlimb motor functions were evaluated at 24, and 48 h using the Tarlov Scale. Besides, spinal cord samples were taken for determination of superoxide dismutase, and catalase activities as antioxidant enzymes, and malondialdehyde as an indicator of lipid peroxidation and xanthine oxidase levels as source hydroxyl radical for biochemical studies. Also, histopathological evaluation was made from cord tissue samples.

**Results:**

The experimental group subjects had better neurological functions than control group subjects. In experimental group; superoxide dismutase and catalase levels increased, while malondialdehyde and xantine oxidase levels decreased as compared with control group. Histopathological examination showed that experimental group had less cell degeneration, hemorrhage, edema and inflammation loss than control group.

**Conclusions:**

This study offers that combined use of N-acetylcysteine and allopurinol might help protect the spinal cord against ischemia/reperfusion injury.

## Introduction

Ischemia-reperfusion (I/R) injury is an important reverse clinical result in a large range of vascular conditions and surgical interventions with the inclusion of aortic diseases. These conditions may cause spinal cord ischemia because of aortic cross clamping, and so violent postoperative complications may improve such as paraplegia [1, 2]. This complication has been featured to provisional or persistent ischemia of the spinal cord caused by deduction of the blood procurementy during aortic cross-clamping [3]. Spinal cord ischemia with resulting paraplegia stays a destructive complication after a repair of aortic aneurysms or dissections. Arterial blood flow failure, trauma of the collaterals of the spinal cord during operation, a low perfusion of distal aorta, a long aortic cross-clamp time, intraoperative proximal hypertension, high pressure of cerebrospinal fluid (CSF), and postoperative hypotension are all regarded as predecessor causes of paraplegia [2, 4]. The incidence of paraplegia has been reported to be as high as 35 % in some series [5–8].

Since a lot of years, although methods such as drug practice and combinations intraperitoneally, intrathecally or intravenously and CSF drainage to increase distal aortic blod flow and perfusion, and reduce spinal cord damage after aorta surgery, none of them could completely prevent spinal cord injury and neurological complications [3, 5, 8, 9].

The most studies showed that release of oxygen free radicals (FRs) by macrophages and neutrophils are associated with I/R injury and free radical generation, lipid peroxidation or influx of calcium into cells can cause neuronal cell death in the spinal cord [8–11]. The tissue-destructive effects of I/R injury are mediated by FRs (H_2_O_2_, O_2_^-^, OH^-^), which damage cellular components and they cause lipid peroxidation of cellular membranes and generate more FRs in a self-propagating cycle, leading to cell death by necrosis [9–11]. In literature some antioxidative and anti-inflammatory agents are used to prevent paraplegia relation to aortic ischemia in rat and rabbit animal models.

We investigated the possible protective effect of N-acetylcysteine (NAC) and allopurinol on neurological, biochemical and histopathological outcomes of spinal cord ischemia and its impact on I/R-induced oxidative damage using a rat infrarenal abdominal aortic clamping model.

## Material and methods

### Animals

A total of 27 male Spraque-Dawley rats weighed between 220 and 370 g were used for the experiment and they were kept in a light-controlled room with a 12:12-h light-dark cycle; temperature (22 ± 0.5 °C) and relative humidity (65 %–70 %) were kept constant. None of them had any neurological disorders before operation. Animals received a standard rat diet and water and libitum. They had not been used in priory another study and they had not been given a drug regularly, in addition they had not a disease, previously. The rats were deprived of food for 12 h before the experiment but had free access to water. Experiments were carried out under steril conditions and antibiotic prophylaxis with cefazolin sodium (30 mg/kg intramuscularly, single preoperative dose) was given. Isotonic NaCl solution was given intravenously at the rate of 3 ml/kg/h. During all experimental manipulations, to prevent the effects of hypothermia and to provide the stability of hemodynamic parameters, the body temperature was maintained at 37.2 °C with a rectal probe. For this, animals were placed on an operating table with thermoregulatory, and were used heat pad.

### Study groups

Twenty-seven rats were randomly allocated into 3 groups. Sham group underwent a surgical procedure similar to the other groups but the aorta was not occluded. This group of animals was used to elicit the effects of anesthesia and operation on the results and also to determine the biochemical parameters of normal spinal cord tissue. In control group, rats underwent a 60-min aortic ischemia without the administration of any drugs to reduce ischemia. In experimental group, subjects were administered NAC (Asist 300 mg ampoule, Hüsnü Arsan Pharmaceuticals) intraperitoneally at a dose of 50 mg/kg 30 min before laparatomy. Besides, allopurinol (Urikoliz 300 mg, Ilsan, Turkey, 50 mg/kg) was administered orally for 2 days before surgical procedure. All cross-clamped groups were re-perfused after spinal cord ischemia of 60 min. Thereafter, all animals received protamine sulfate (1 mg/kg intravenously) to antagonize the effects of heparin.

### Operative technique and I/R procedure

The rats were anesthetized with intramuscular injection of 15 mg/kg ketamine hydrochloride (Ketalar; Pfizer, Istanbul, Turkey) and 2 mg/kg xylazine hydrochloride (Rompun, Bayer, Turkey) before the surgical procedure. The rats were shaved from abdomen to leg. The surgical area was painted with batticon. Surgical area was cleaned and draped. Intraperitoneal cephalosporine (10 mg/kg) was administered before skin incision. After steril surgical preparation, a midline laparotomy was made and the intestines were taken out by deflecting them to the right and covering them with warm and wet compresses to decrease the loss of heat and fluid. After the retroperitoneal area was opened, the abdominal aorta and inferior vena cava were identified and isolated. The maintenance of anesthesia was established with intermittent delivery of ketamine, without endotracheal intubation and mechanical ventilation. 400 IU/kg of heparin was administered intraperitoneally to all animals immediately before the procedure. Spinal cord ischemia was created by clamping the aorta just below the renal vein with an atraumatic microvascular clamp (vascustatts II, midi straight 1001–532; Scanlan Int., St. Paul, MN, USA) 3 min after heparin administration. Another clamp was placed above the aortic bifurcation to occlude the iliac collateral circulation. During procedure, temperature probe was inserted into the rectum. Body temperature was maintained close to 37 °C using a heated operation table. The systemic blood pressure was measured as 70–80 mmHg in the aorta. Electrocardiographic monitoring was performed on all animals throughout the experiment. The heart rate was maintained at 176–190 beats/min. After experimental procedure, all animals received protamine sulfate (1 mg/kg intravenously) to antagonize the effects of heparin. After bleeding was controlled, 10 ml of warm ringer lactate solution was given intraperitoneally. The muscles of the abdomen and skin were closed in a routine manner with 3–0 silk sutures. Prophylactic antibacterial therapy was carried out with the intramuscular administration of Cefazoline Na (15 mg/kg/day; Mustafa Nevzat AS, Istanbul, Turkey) preoperatively and postoperatively. For postoperative analgesia, morphine HCl (400 mcg/kg/day, i.m., Galen AS, Istanbul, Turkey) was given to all subjects.

### Evaluation of neurological status

After reperfusion, two of the study authors who are blinded to the groups of the animals have evaluated the hindlimb motor function at 24 and 48 h according to Tarlov Scale [12]. Grade 0: complete paralysis, Grade 1: minimal movement in articulations, Grade 2: unable to stand without support, Grade 3: able to stand by themselves, Grade 4: poor jumping, Grade 5: complete recovery.

### Sacrifice of subjects

After neurological evaluation, the rats were re-anesthetized with a high dose of ketamine (100 mg/kg). The spinal cord tissue was extracted and fixed in buffered formalin for 10 days to analyze the tissue level of SOD, CAT, MDA, and XO and histopathological examination. All animals were sacrificed after the procedure.

### Evaluation of pathological specimens

Spinal cord specimens were fixed in 10 % neutral buffered formaldehyde solution. After dehydration procedures, the samples were blocked in paraffin. 5-μ sections were cut by a microtome and stained with hematoxylin-eosin. The mounted slides were examined under a light microscope (Nikon Microscope ECLIPSE E600W, Tokyo, Japan) at 200 x magnification (cell degeneration, edema, inflammation, and congetion). The distribution of cell degeneration, intensity of edema, inflammation and hemorrhage in the spinal cord were semi-quantitatively scored from 0 to 4 (no, low, moderate, high and very high, respectively).

### Biochemical examination

SOD, CAT, XO, and MDA were detected in muscle tissue cuts. Each tissue was stocked in a separate bowl at −80 °C till analysis. Tris tampon of 10 ml was added into each one gram of frozen tissues. Homogenates are to be centrifuged at 10.000 × G for 10 min after homogenization. Supernatants were kept in stock at −80 °C till analysis. Analysis of tissue samples was carried out spectrophotometrically as below. Results were expressed as units per miligram protein for SOD, CA T nanomoles per milligram for MDA and miliunits per milligram for XO.

### Tissue SOD assay

This method is based on the inhibition of nitroblue tetrazolium (NBT) reduction by the xanthine–xanthine oxidase (XO) system as a superoxide generator using the Yi-Sun method [13]. The study solution was prepared by mixing xanthine (0,3 mmol/l), ethylenediaminetetra-acetate (EDTA) (0.6 mmol/l), NBT (0.15 mmol/l), sodium carbonate (Na_2_CO_3_) (400 mmol/l), and bovine serum albumin (1 g/l). The study solutions of 2850 μl, 100 μl supernatant, 100 μl distilled water and 50 μl XO (5 U/l) were incubated for 25 min at 20 °C. After 30 s, absorbance was recorded. One unit is the amount of SOD that inhibits the rate by 50 %.

### Tissue CAT assay

Catalase activity was assayed according to the methods of Cohen et al [14]. To a 100 μL aliquot of tissue extract, ethanol was added to a concentration of 0.17 mol/L (10 μL ethanol/mL) and samples were incubated in an ice bath for 30 min. After 30 min, 10 % Triton X–100 was added to a final concentration of 1 % and samples were kept at room temperature. Reactions were performed at room temperature. The enzyme-catalysed decomposition of H_2_O_2_ was measured. In a tube containing 200 μL phosphate buffer and 50 μL tissue extract, 1 mL of 6.0 mmol/L H_2_O_2_ (in phosphate buffer) was added and mixed thoroughly. The reaction was stopped after exactly 3 min by the addition of 100 μL of 6 mol/L H_2_SO_4_. The excess H_2_O_2_ was measured by reacting it with a standard excess of KMnO_4_ and then measuring the residual KMnO_4_ spectrophotometrically at 480 nm within 30–60 s using 1,0 absorbance unit for standard KMnO_4_.

### Tissue MDA assay

Whole blood MDA (as an important indicator of lipid peroxidation) levels were measured according to the method of Jain et al [15]. The principle of the method is based on the spectrophotometric measurement of the color that occurs during the reaction of thiobarbituric acid with MDA. The concentration of thiobarbituric acid reactive substances (TBARS) was calculated by the absorbance coefficient of the malondialdehyde–thiobarbituric acid complex and expressed in nmol/ml.

### Tissue XO assay

Xanthine oxidase activity was determined spectrophotometrically by the method of Hashimoto [16] based on the formation of uric acid from xanthine at 293 nm.

### Statistical analysis

Statistical analysis and calculations were performed by using SPSS 15 for Windows (SPSS Inc., Chicago, IL, USA). All the results were obtained as mean ± SEM for each study group. The significance of differences between the groups was assessed using the Non-parametric analyses with Mann-Whitney *U*-test (one-way analysis of variance (ANOVA) and Tukey’s posttest), and the Kruskal-Wallis test was used to compare group medians for histopathological. Two groups were compared based on the parameters of the Tarlov scoring. A value of p less than 0.05 was considered significant. A value of p less than 0.05 was considered significant.

### Ethical compliance

All studies were performed in the Laboratory of Experimental Animal Research and Practice of A.U. Medical Faculty. The experiment was performed in compliance with the Principles of Laboratory Animal Care formulated by the National Institutes of Health. The experiment and animal care protocol, and all procedures were approved by the Local Ethics Committee in Animal Experiments.

## Results

### Neurological evaluation

**Fig. 1:**
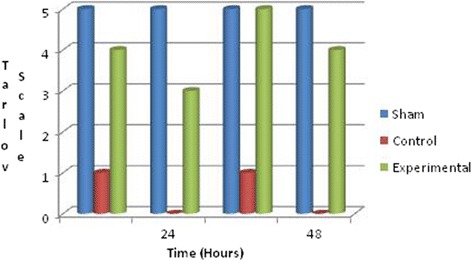
Neurological status of animals in each group according to the Tarlov Scoring System

All animals were evaluated and graded based on the Tarlov Scale, at 24 and 48 h after reperfusion. In control group, seven rats were grade 0, one was grade 1, and one was grade 3. In experimental group, no rat had total paraplegia. One of them was observed as having grade 3, three grade 4, and five grade 5. The rats in experimental group had better neurological functions than those in control group (*P* < 0.01) (Fig. 1) (Table 1).

**Table 1:** Number of animals in each group according to the Tarlov Scale

Tarlov Scale (Grade)	Sham group (n = 9)	Control group (n = 9)	Experimental group (n = 9)
0	0	7	0
1	0	1	0
2	0	1	0
3	0	0	1
4	0	0	3
5	9	0	5

Grade 0: complete paralysis, Grade 1: minimal movement in articulations, Grade 2: unable to stand without support, Grade 3: able to stand by themselves, Grade 4: poor jumping, Grade 5: complete recovery

### Histopathological analysis

**Fig. 2:**
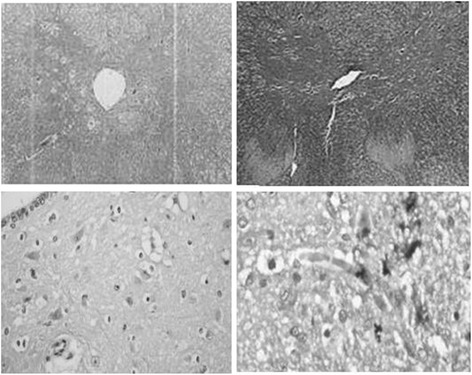
Hematoxylin and Eosin x 200 staining of the medulla spinalis tissue. Results of histopathological analysis of all experimental groups. A spinal cord region at 60 min postischemia shows widespread hemorhage, edema, cell degeneration, and inflammation (H&E, ×200)

Following 48 h reperfusion, in the histopathological evaluation of spinal cord sections, in statistically significant hemorrhage areas, inflammation, edema, and cell degeneration observed in control group when compared to experimental group. In sham group, histopathological evaluation showed normal data; therefore the results of group sham have not been included in the table because a significant histopathological difference was observed between control and experimental groups (*p* < 0.05), (Table 2, Fig. 2).

**Table 2:** Comparison of histopathological changes between control and experimental groups

	Control Group		Experimental Group		p
	Mean	SD	Mean	SD	
Inflammation	3.16	0.88	1.01	0.91	0.002
Edema	3.92	0.68	0.99	0.84	0.001
Cell Degeneration	2.55	0.97	0.87	0.77	0.005
Hemorrhage	2.88	0.64	1.11	0.87	0.007

Parameters were scored semi quantitatively from 0 to 4 (0: no; 1: low; 2: moderate; 3: high; 4: very high

There was a significant difference in terms of pathological parameters between experimental and control group as histopathological scores. Figure 2 shows markedly hemorrhage edema, cell degeneration, and inflammation in control group after 48 h reperfusion. In experimetal group, hemorrhage, edema, cell degeneration, and inflammation decreased markedly compared to control group.

(*P* < 0.05) (Table 2, 3) (Fig. 2). The histopathological findings showing the spinal cord in drug group with more healthy cells and less cell degeneration. In the experimental drug group, a light microscopic examination of the spinal cord region reveals both healthy cells and a significant decrease in neuronal destruction.

**Table 3:** Histopathologic results of the medulla spinalis tissue

	Grade	Control group	Experimental group	*P* value
		Number (%)	Number %	
Cell Degeneration	0	0 (0)	7 (77.8)	0.0001
	1	0 (0)	1 (11.1)	
	2	1 (11.1)	1 (11.1)	
	3	2 (22.2)	0 (0)	
	4	6 (66.7)	0 (0)	
Edema	0	0 (0)	6 (66.7)	0.0003
	1	0 (0)	1 (11.1)	
	2	0 (0)	1 (11.1)	
	3	4 (44.4)	1 (11.1)	
	4	5 (55.6)	0 (0)	
Hemorrhage	0	0 (0)	5 (55.6)	0.0009
	1	1 (11.1)	2 (22.2)	
	2	1 (11.1)	2 (22.2)	
	3	2 (22.2)	0 (0)	
	4	5 (55.6)	0 (0)	
Inflammation	0	0 (0)	8 (88.9)	0.0001
	1	2 (22.2)	1 (11.1)	
	2	1 (11.1)	0 (0)	
	3	2 (22.2)	0 (0)	
	4	4 (44.4)	0 (0)	

### Biochemical analysis

**Fig. 3:**
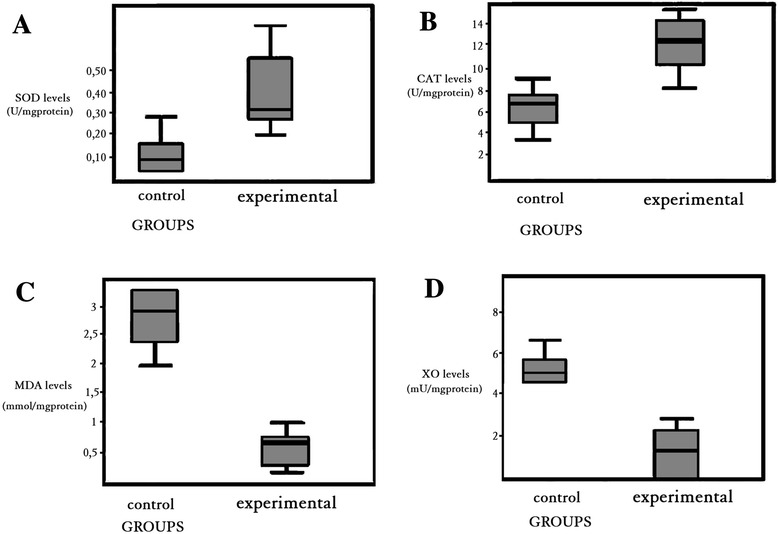
Effects of NAC and allopurinol on spinal cord tissue. Between groups antioxidant enzyme levels. **a**, **b** Increased SOD and CAT levels in group experimental in comparison to control group. **c**, **d** Decreased MDA and XO levels in experimental group in comparison to control group. *SOD*, superoxide dismutase; *CAT*, catalase; *MDA*, malondialdehyde; *XO*, xanthine oxidase

SOD, CAT, MDA, and XO levels were measured in spinal cord tissue specimens after 48 h reperfusion. SOD and CAT levels increased in the experimental groups compared to control group (from 0,10 ± 0,07 to 0,33 ± 0,05 for SOD; from 6,96 ± 0.90 to 12,52 ± 0,80 for CAT), and and it was significant as statistically (*p* < 0.05) (Fig. 3a and Fig. 3b)*.* XO levels in the spinal cord were found to be higher in the control group. However, in treatment groups lowered (from 5,11 ± 0,55 to 1,71 ± 0,44) the levels of XO Tissue MDA levels were decreased (from 2,92 ± 0,09 to 0,61 ± 0,08) in the treatment group compared to the control group. The results of SOD, CAT, MDA, and XO assays are shown in Fig. 3a–d.

## Discussion

This study proposes that prophylactic administration of *N-*acetylcysteine and allopurinol combination can reduce ischemia-reperfusion injury of spinal cord in an aortic occlusion model of rats.

Paraplegia is an rare but destructive complication, which visibles after thoracic or thoracoabdominal aortic surgery due to low perfusion pressure in spite of miscellaneous surgical and pharmacological interferences [17]. The reported ratio of paraplegia intervals from 3.8 % to 17.6 % [18]. Spinal cord injury may complicate the repair of wide thoracoabdominal aortic diseases because of ischemic injury to the spinal cord that results from the deduction of intercostal and lumbar arterial blood flow, and postoperative neurological deficiencies are watched in a lot of cases [3, 19].

Arterial vascularization of the spinal cord is similar in rat and man. In addition, a rat model ensures important advantage for animal studies such as continuation of normothermia along the operation and the development of important paraplegia after procedure [20, 21]. So, many studies have been designed to determine the protective effects of distinct pharmacological substances during ischemia of the spinal cord in rat models as in our study. The cause for the use of rats as experimental animals in this study was that these animals have previously been commonly used in similar experimental models in which the main blood flow of the spinal cord originated from the distal aorta, and the suitability of laparotomy for an experimental study has been shown elsewhere [19, 22, 23]. Many methods can be used to minimize paraplegic complications; they include hypothermia, solitary clamping, arterial shunts, the use of antioxidant drugs [1, 6, 24, 25].

The best method to protect the spinal cord from damage during ischemia/reperfusion in thoracoabdominal aortic surgery is to apply the clamp at a point distal of these areas, if possible, while observing both the anterior spinal artery and arteria radicularis magna, in order to maintain a short clamping period and keep the distance between the clamping and reimplantation of intercostals as much as possible [24–26]. Kouchoukos [27] reported a correlation to exist between paraplegia and the time of clamping and the length of harvested aorta. However, the reimplantation of the intercostals to the graft as far as possible is one of the best methods of protection, and especially all possible intercostals and lumber arteries between T6 and L1 should be reimplanted to the graft as far as possible. Svensson et al [24] identified these arteries by a special method perioperatively by which the incidence of paraplegia after their operations decreased. Yamada et al [28] preoperatively revealed the ASA, ARM, and critical intercostal arteries by magnetic resonance angiography. They obtained notable results in the protection of the cord by reimplanting the determined critical arteries to the int erposed aortic graft.

In literature there are several studies that evaluating the protective effects of the different pharmacological agents and their combination (simvastatin, pentoxifylline, dantrolen Na, vitamins, ilioprost and N-acetylcysteine combination, etc.) on the spinal cord I/R injury [29, 30]. All researchers agree that many agents may partially prevent reperfusion injury, but none have been found to be sufficient by itself; it must be combined with other agents and protective surgical methods [28, 31, 32]. We examined that the NAC and allopurinol combinations decreased I/R damage, both histopathological and biochemical. Our literature search did not produce any study showing the efficacy of NAC and allopurinol in a spinal cord ischemia/reperfusion model.

Free radicals (FRs) are normal by-products of cellular metabolic processes. The human body has a complex antioxidant defense system that includes the antioxidant enzymes (SOD and CAT) and nonenzymatic antioxidant components such as glutathione, a-tocopherol, ascorbic acide, and b-carotene. These prevent the initiation or propogantation of free radical chain reactions. Post-ischemic reperfusion injury is associated with the generation of FRs which damage cellular components and initiate the lipid peroxidation process. In many studies, antioxidant activity was tried to be shown through biochemical enzyme studies in addition to histopathological studies. SOD can scavenge the superoxide radicals and can play an important role in preventing oxidative injury and protecting the cells, metabolizing O_2_ against FR’s harmful effects. This inhibits the peroxidation of lipids, and the intracellular superoxide activities decrease. If ischemia occurs for a long period of time, the SOD activities will be lower, because it has a short lifetime. Some authors have reported the tissue SOD activities to be significantly higher in the drug groups of the I/R model. When oxidant stres increases in organism, SOD enzyme levels increase [16, 33, 34]. The function of CAT is to divide H_2_O_2_ into O_2_ and H_2_O by participating in the reaction with H_2_O_2_, thereby preventing the formation of OH^-^ radicals, which are more toxic [35, 36]. Criado found that CAT enzyme levels increased in 30 min after I/R damage [35]. In our study, the SOD and CAT levels significantly increased in experimental group which received antioxidant agents.

Lipid peroxidation has been reported as an important contributor to the loss of cell function under oxidative stress conditions. The end production of lipid peroxidation includes aldehydes, hydrocarbon gases, and MDA [37]. MDA is important and the most commonly used indicator of lipid peroxidation, and its level increases in tissues when they are exposed to oxidative stress. Recent studies have shown that lipid peroxidation increases in I/R injury of the spinal cord and serum following aortic clamping [38, 39]. Belboul et al. [40] showed an increase in the MDA levels in patients who undergo coronary artery surgery, following 30 min of reperfusion. After removing the clamps, the MDA levels increased for a second time. Lu et al. [41] reported increased MDA levels at all times of reperfusion in their study. In our study, the MDA levels had significantly increased after ischemia reperfusion in the control group, because of the high level of hydroxyl radicals. In the experimental group, the levels were found have decreased, which may show the effects of antioxidant medication on limiting ischemia reperfusion injury. XO is the first known source of superoxide radicals. XO plays an important role in I/R injury. Xanthine dehydrogenase, the natural form of it, cannot produce superoxide radicals [42]. Xanthine dehydrogenase easily transforms into an oxidase form because of sulfhydryl oxidation or practical proteolysis, thus developing during ischemia. This transformation from xanthine dehydrogenase to oxidase has been shown in experimental studies [43–46]. Hammerman showed that lipid peroxidation was prevented in the group together with the decrease in XO activity [47]. Most studies have demonstrated XO activity to significantly decrease in the animals of the group that received antioxidant agents [48]. In our study, there were significant differences in the levels of XO between experimental groups and control group*.*

*N*-Acetylcysteine has a mucolytic and antioxidant effect. It obtains oxygenation of tissues and can protect the spinal cord partially from reperfusion injury [31, 49]. Many authors have reported the benefits of antioxidant administration in various models of ischemia/reperfusion. *N*-Acetylcysteine is a good candidate, as it is well tolerated in humans and has few side effects. In addition, the cords of animals with no motor function deficits showed only minimal cellular infiltrates in the gray matter, and there was a good preservation of nerve cells. It also showed a protective effect on the spinal cord and resulted in a highly significant recovery of spinal cord function [2, 31].

Allopurinol, a specific inhibitor of the enzyme XO, blocks the synthesis of xanthine from hypoxanthine and therefore avoids the formation of the free radical superoxide. The studies showed that it is decrease the level of FRs production and reduce the tissue injury associated with I/R injury [50, 51]. It is not only a potent inhibitor of XO but may also be an agent that improves ischemia-induced mitochondrial dysfunction [51, 52].

For neurological group evaluation, we used the Tarlov Scale [12]. Ilhan et al [6] evaluated their subjects neurologically using the Tarlov Scale. Although there are a lot of neurological evaluation methods, we used Tarlov’s Scale, since it is simple and more practical 48 h after reperfusion on spinal cord ischemia model [2, 25, 53]. The scores changed from the subjects with no neurological findings (Tarlov’s Scale grade 5) to complete paralysis (grade 0). In our study, by comparing the two groups statistically according to the Tarlov scores, the scores in experimental group were demonstrated to be significantly higher than those in control group.

In our study, there were no histopathological changes in sham group. Histopathological analyses of spinal cord samples from control and experimental groups showed that the incidence of cell degeneration, edemas and inflammation and, hemorrhage as evidence of a bad prognosis, was lower in the samples from experimental group compared to those from control group.

## Conclusions

This study suggests that in rat model of spinal cord injury prophylactic use of *N*-Acetylcysteine and allopurinol combination may reduce ischemic damage of spinal cord and may provide beter neurological outcome. The combination of these drugs with other known standard treatments and protective surgical techniques may decrease morbidity in high-risk patient groups that undergo aortic surgery, and prevents reperfusion injuries by eliminating oxygen radicals and inhibiting lipid peroxidation. Administration of NAC and allopurinol spinal cord I/R decreased MDA and XO levels and increased in SOD and CAT enzyme activities in the spinal cord and this result was suggested with hystopathological results. Efficacy of different dosage strategies and different administration durations of these drugs should be evaluated in further studies.
